# Gene-Metabolite Interaction in the One Carbon Metabolism Pathway: Predictors of Colorectal Cancer in Multi-Ethnic Families

**DOI:** 10.3390/jpm8030026

**Published:** 2018-08-06

**Authors:** S. Pamela K. Shiao, James Grayson, Chong Ho Yu

**Affiliations:** 1 Medical College of Georgia, Augusta University, Augusta, GA 30912, USA; 2 Hull College of Business, Augusta University, Augusta, GA 30912, USA; jgrayson@augusta.edu; 3 Department of Psychology, Azusa Pacific University, Azusa, CA 91702, USA; cyu@apu.edu

**Keywords:** metabolites and genes, one carbon metabolism pathways, colorectal cancer, generalized regression with validation, diverse ethnic groups

## Abstract

For personalized healthcare, the purpose of this study was to examine the key genes and metabolites in the one-carbon metabolism (OCM) pathway and their interactions as predictors of colorectal cancer (CRC) in multi-ethnic families. In this proof-of-concept study, we included a total of 30 participants, 15 CRC cases and 15 matched family/friends representing major ethnic groups in southern California. Analytics based on supervised machine learning were applied, with the target variable being specified as cancer, including the ensemble method and generalized regression (GR) prediction. Elastic Net with Akaike’s Information Criterion with correction (AICc) and Leave-One-Out cross validation GR methods were used to validate the results for enhanced optimality, prediction, and reproducibility. The results revealed that despite some family members sharing genetic heritage, the CRC group had greater combined gene polymorphism-mutations than the family controls (*p* < 0.1) for five genes including *MTHFR* C677T, *MTHFR* A1298C, *MTR* A2756G, *MTRR* A66G, and *DHFR* 19bp. Blood metabolites including homocysteine (7 µmol/L), methyl-folate (40 nmol/L) with total gene mutations (≥4); age (51 years) and vegetable intake (2 cups), and interactions of gene mutations and methylmalonic acid (MMA) (400 nmol/L) were significant predictors (all *p* < 0.0001) using the AICc. The results were validated by a 3% misclassification rate, AICc of 26, and >99% area under the receiver operating characteristic curve. These results point to the important roles of blood metabolites as potential markers in the prevention of CRC. Future intervention studies can be designed to target the ways to mitigate the enzyme-metabolite deficiencies in the OCM pathway to prevent cancer.

## 1. Introduction

Chronic inflammation is a major risk factor for colon and rectum health that underlies the development of colorectal cancer (CRC), with CRC being preventable by modifying lifestyle interventions for human ecological development [1,2,3,4,5,6]. Well-defined lifestyle interventions may improve cancer treatment effects, prevent cancer progression and increase survival through epigenetic mechanisms with gene-environment interactions [1,4,5]. Most causes of CRC are related to environmental and lifestyle factors, while about 30% of CRC risk is inheritable, with 5% being highly aggressive in cancer progression for metastatic penetrance [7,8,9]. Hence, the most common risks for CRC are preventable by cultivating healthy lifestyles to help keep the human epigenetic environment free from cancers. Hyperhomocysteinemia is highly prevalent in patients with inflammatory bowels [2,10,11], and might be caused by either decreased absorption or increased requirements for folate (vitamin B9) and other related B vitamins (B2 (riboflavin), B6 (pyridoxine), and B12 (cobalamin)) that are all required for one-carbon metabolism (OCM) pathway and homocysteine metabolism [12,13,14,15,16,17]. Elevated homocysteine level is an independent predictor for all-cause mortality [18,19] and it compromises health of all organ systems [20,21,22,23], affecting epigenetic changes for DNA synthesis and healthy living. For each 5 μmol/L homocysteine increment, the risk of mortality increased by 32%, and the risk of heart disease increased by 52% [18]. When gene mutations in the OCM pathway occur, such as with the *methylenetetra-hydrofolate reductase (MTHFR)* C677T (rs 1801133) polymorphism, there is a deficiency in the methyl-folate enzyme and the activity in the OCM pathway is impaired [13,14,23,24,25,26]. However, an increase in methyl donors such as vitamin B2, B6, B9, B12, or methionine, may help compensate for the deficiency of the enzyme metabolites in OCM pathways during DNA methylation, synthesis and repair, thus preventing carcinogenesis [27,28]. Deficiency in B12 levels is commonly observed in cancer patients receiving advanced cancer and radiation treatments which is associated with elevated methylmalonic acid (MMA) levels [29,30,31,32].

We examined five genes in the OCM pathway, including two loci of *MTHFR* gene polymorphisms, C677T (rs1801133) and A1298C (rs1801131), both are associated with MTHFR enzymatic deficiency resulting in increased homocysteine concentrations [33,34]. *MTHFR* polymorphism leads to missense/loss of function mutation of 677C→T, resulting in a thermolabile enzyme variant that has a reduced catalytic activity of 35% for 677 CT and 70% for 677 TT variants, and of nucleotide 1298A→C, resulting in 15% decreased MTHFR activity for 1298 AC and 30% for 1298 CC variants [35,36]. We also investigated three additional genes in the pathway: *Dihydrofolate reductase (DHFR)* 19 base pair (19bp) (rs70991108) which converts folic acid into methylenetetrahydrofolate (MTHF) or methyl-folate as usable folate form [37,38], *methionine synthase* (*MTR* A2756G, rs1805087) in the methylation cycle, and *methionine synthase reductase* (*MTRR* A66G, rs1801394) which converts/recycles homocysteine back to usable methionine for the methylation cycle [39,40,41,42]. Together, these five genes play critical roles in the OCM methylation pathways for biological processes in sustaining human health, and polymorphism-mutations of these genes would lead to lost functions for the methylation process.

Key methyl-donors related to these genes include folate (vitamin B9) and vitamin B12, that play an integral role in the phenotypic expression of *MTHFR* and related gene mutations in the OCM methylation pathways [33,34,35,36]. The methyl-donors could compensate for the deficient enzyme-metabolites in the methylation pathways resulted from the loss-of-function gene mutations in the OCM pathway. Therefore, the purpose of this study, extending from a previous study on gene-environment interactions [43], was to examine the key metabolites and genes in the OCM pathway that may affect the risk associations with CRC, and the related factors affecting the risks of CRC. In this study, we used machine learning based analytic methods including the bootstrap ensemble method [44,45,46,47], as well as generalized regression (GR) in addition to the baseline logistic regression (LR) models, for predictive modeling to cross-validate the results [48,49,50,51].

## 2. Materials and Methods

### 2.1. Study Population and Setting

The study methods were reported before [43,52] and are summarized in the following. We included 30 participants, 15 CRC cases and 15 matched family/friend controls by accessing the California Cancer Registry (CCR) database and other cases through referrals from the community where the study was conducted. The designated Human Subjects Institutional Review Boards (IRB) from the local educational institutions and the California State Committee for the Protection of Human Subjects (CPHS 12-12-1007) approved the project [43,52]. With the approved study procedures, the qualified participants were recruited. The participants were interviewed on campus or in their homes.

### 2.2. Demographic Data

Demographic data included lifestyle and dietary status [43,52,53], family history, functional capacities using the items included in the 1999–2012 National Health Interview Survey [54] and the family pedigrees from the Coalition for Health Professional Education in Genetics [55].

### 2.3. Genotyping and Matabolites Data

Gene polymorphism and metabolite analysis were performed at the Center of Metabolomics, Baylor Scott & White Research Institute, Dallas, TX, USA. Data sent to the laboratory were de-identified for subjects. Laboratory staff members were blinded to the case control and other status of the samples to enhance the objectivity of laboratory analyses. The specimens were stored on ice and sent in containers with dry ice via express mail to the laboratory following data collection. Once arrived at the laboratory, specimens were kept frozen in deep freezer at −80 °C freezer until analysis. Plasma total homocysteine was determined by liquid chromatography–electrospray ionization tandem mass spectrometry (LC–ESI/MS-MS) as previously described [56]. Plasma *S*-adenosylmethionine (SAM), *S*-adenosylhomocysteine (SAH), betaine, choline, methionine, cystathionine were quantified by LC-ESI/MS/MS methods described previously [57,58], and modified to include asymmetric dimethylarginine (ADMA), and symmetric dimethylarginine (SDMA). Serum 5-MTHF was quantified by LC-ESI/MS-MS using previously described methods [59]. Plasma MMA was determined by LC-ESI/MS-MS as previously described [60]. The inter-assay coefficient of variation for all analytes were below 15%. The analysis of metabolites was performed on 4000 QTrap and 5500 QTrap mass spectrometry instruments (Sciex, Framingham, MA, USA) coupled to LC systems (Shimadzu, Columbia, MD, USA) with data collected and processed using Analyst Software Version 1.6.2 (Sciex, Framingham, MA, USA).

Genotyping procedures were described elsewhere earlier [61,62]. Briefly, genomic DNA was isolated from salivary samples using the SK-1 swab and Isohelix collection tubes with dry capsules (Boca Scientific, Boca Raton, FL, USA), and/or from peripheral blood samples using the Qiagen Blood DNA Kit (Qiagen Inc., Valencia, CA, USA). The Taqman technique [61] was used for genotyping of the gene polymorphisms using allele specific fluorescent probes with a StepOnePlus™ Real-Time polymerase-chain reaction System (Thermo Fisher Scientific, Waltham, MA, USA). Quality control was strictly conducted with four duplicate positive controls and four negative controls loaded in each of 96-well plates. Additionally, genotyping assays were repeated with 10% of the samples that were duplicate with salivary and blood samples, and genotyping results were in 100% agreement for the repeated tests. In addition to the four gene polymorphisms (*MTHFR* C677T and A1298C, *MTR* A2756G, and *MTR* A66G) that were presented for the CRC cases [39,42], and in numerous meta analyses [12,13,14,15,16], we included *DHFR* 19 bp deletion as an additional gene in the folate-metabolism pathway. *DHFR* 19 bp in the folate methylation pathway has not been presented for the CRC cases in various ethnic groups before. The total gene mutations from 5 genes were computed together, with possible ranges of 0–10, with scores of ‘1’ for heterozygous and ‘2’ for homozygous polymorphism per each gene. The total gene polymorphism rates of the 5 chosen genes in the folate methylation pathways could range from 0 to a possible maximum score of 10 if each of the 5 genes had homozygous polymorphisms. MTHFR enzyme deficiency was calculated by combining the loss of enzyme functions from *MTHFR* C677T (loss of 35% for each of the two T polymorphic alleles) and *MTHFR* A1298C (a loss of 15% for each of the two C polymorphic alleles), a composite score of both *MTHFR* C677T and *MTHFR* A1298C polymorphisms [43,63].

### 2.4. Data Analysis

Machine learning based analytics were employed in JMP Pro 13 (SAS Institute, Cary, NC, USA) [64,65]. Unlike conventional statistics, in machine learning the sample is randomly partitioned into subsets, and the algorithm repeats the same analysis in different subsets, in order to learn from different examples for model improvement. Machine learning could be supervised or unsupervised [66]. In this study we employed supervised machine learning because the target variable is specified. The analytics and rationales have been reported earlier [43,52] and are summarized in the following. We used bootstrap forest, also known as bagging (i.e., bootstrap aggregating), which is one of the most popular ensemble methods [44,45,46,47]. The ensemble methods are based on the logic of resampling, which is a well-known remedy for small-sample studies [67,68]. For example, while developing the bootstrapping method in 1983, Diaconis and Efron had only 15 observations [68]. In resampling, the sample is treated as the virtual population and then different subsets are randomly drawn from the sample for multiple analyses. Bias can be observed and corrected by such repeated analyses on random subsets [69]. This approach is superior to conventional regression modeling because ordinal least square regression or logistic regression (LR) analyses tend to yield an overfitted model. Numerous studies have confirmed that the ensemble approach outperforms any single model, such as regression or univariate statistics [70,71,72]. In addition, conventional statistical procedures are limited by the sample size. If the number of parameters to be estimated exceeds the degrees of freedom, the regression model would be highly unstable. When different models are generated by resampling, inevitably some are high bias model (underfit) while some are high variance model (overfit). In the end, the ensemble cancels out these errors. Specifically, each model carries a certain degree of sampling bias, but finally the errors also cancel out each other [71]. Our strategy was to identify the most influential predictors within the categories of genetic factors, metabolites, and demographic/lifestyle factors as indicated by health metrics. To select the most influential predictors within each category, we used the criteria of column contribution and variable importance. The column contribution is presented using the *G*^2^ statistics, which is derived from the conventional likelihood ratio *X*^2^ statistic, as *X*^2^ is a test of goodness-of-fit between the expected count and the actual account. Individual predictors were selected by using the decision tree methods to build models and then from the rank order of column contributions selecting the most influential variables using the bootstrap forest method [46,47].

The most significant variables and potential interactions were visualized using the interaction profilers for bi-variate interactions of the three categories of variables, and the final set of significant variables were selected for the tested models. The prediction profiler and interactive profiler can be used to visualize the direction of association between two parameters (a predictor or factor with the outcome variable of status in profiler) or among three parameters (set of interactive variables with non-parallel distribution in addition to the outcome status in the interactive profiler). The visualization of the profiler and interactive profiler will enable the analyst to visualize and account for the interactions of various factors.

We used GR to obtain a smaller prediction error [64]. Generalized regression is also known as penalized regression, meaning that the variable selection process penalizes complexity. As the name implies, the modeling process penalizes complicated models to avoid overfitting. To get the optimal model, the algorithm imposes a penalty on the model when redundant predictors are included. With the machine learning approach, these models included a random validation dataset to yield more reliable prediction. Hence, compared with conventional regression modeling, GR tends to yield an optimal model. The index showing complexity is Akaike information criteria (AIC) or AIC with correction (AICc) [72,73,74], developed by Hirotsugu Akaike [75,76]. In this approach the simplest model tends to be the best one. Specifically, AIC is a fitness index for trading off the complexity of a model against how well the model fits the data. Increasing the number of free parameters to be estimated improves the model fitness, however, the model might become unnecessarily complex. To reach a balance between fitness and parsimony, AIC not only rewards goodness of fit, but also includes a penalty against over-fitting and complexity. Hence, the most optimal model is the one with the lowest AIC value. Since AIC attempts to find the model that best explains the data with a minimum number of free parameters, it is considered an approach favoring simplicity. In this sense, AIC is better than *R*^2^ and adjusted *R*^2^, which always go up as additional variables enter in the model, favoring complexity. However, AIC does not necessarily change by adding variables. Rather it varies based upon the composition of the predictors and thus it is a better indicator of the model quality [77]. AICc converges to AIC as the sample size gets larger and larger. AICc should be used regardless of sample size and the number of parameters. We examined model quality using the misclassification rate (smaller is better), AICc, and the area under the receiver operating characteristic (ROC) curve (AUC).

When developing a GR model for a predictive model, the first type of model presented in JMP Pro 13 is a logistic regression (LR) model because the default estimation method is an LR. After this default method, other model launches can be pursued by choosing a variety of estimation methods (Least absolute shrinkage and selection operator (Lasso), Elastic Net and others) and associated validation methods (a validation column, minimum AICc, leave-one-out (LOO) validation and others, [78]). Both AICc validation and LOO cross-validation methods are effective methods for small data sets [79]. In effect, the default LR method could be characterized as an explanatory model, whereas the other GR estimation methods might best be characterized as a predictive model. An explanatory model is typically used to explain the association between the model parameters and the model response to test causal hypotheses, using a predictive model, for predicting future observations [80]. The predictive model using GR will pursue methods to shrink coefficients towards zero in part to guard against overfitting the model. Unlike linear least squares in estimating the unknown parameters in a linear regression model, GR could simply zero out certain unused predictors [81]. In traditional statistics, usually one model is used to fit the data, and thus the probability is nothing more than an approximation based on sampling distributions, which are open-ended (the two-tails of the curve never touch the *x*-axis). In this case, the *p* value at most could only be 0.9999, but not exactly one. However, when all permutations are exhausted, such as what was done in an exact test, the probability could be exactly one. In a similar vein, GR exhausts different paths to find the best model. When the full model has a mixture of important and unused predictors, the *p* value cannot be one. However, when the data could be perfectly described by the restricted model resulting from path searching, the probability of observing the data could be one.

## 3. Results

### 3.1. Characteristics of Study Participants

We attempted to match the CRC and family groups on various demographic factors for this family-based study. During data visualization within each of the CRC and family control groups, we identified clinical factors that may affect the outcomes. That is, additional chronic health conditions such as diabetes and chronic inflammatory diseases or advanced cancer stage were recognized within the two groups, hence, we explored the potential differences among the four groups with two groups within each group. Table 1 presents the comparisons of key demographic and lifestyle health metrics [53] among these four groups. We used non-parametric tests and non-parametric post-hoc tests to identify differences on these parameters among the four groups. Parameters that were significantly different between the control and cancer groups included age and gender (both *p* < 0.05). The family control group had a younger age because many of the available family members were the offspring of the cancer patients. As seen in Table 1, the advanced disease groups had older ages than the control group without health conditions (*p* < 0.05 for two of the post-hoc group comparisons, CRC with advanced inflammatory health issues being oldest). For dietary healthy eating, the advanced cancer group ate the least portion servings of vegetables and fruits (*p* < 0.1, and *p* < 0.05 for post hoc tests on the difference between early stage and advanced cancer stage groups). As this was a proof-of-concept study, additional adjustment of *p*-values for multiple testing was not used for the exploratory analyses of related factors.

The demographic/lifestyle factors were compared across the racial–ethnic subgroups (Supplementary Table S1). The results showed that the Caucasian and African American samples presented higher body mass index (BMI) than Asians, and Caucasians took more whole grains than the Hispanic and Asian samples (all *p* < 0.05). We present the distributions of the genotype alleles for five genes in the OCM pathway for the four groups (Table 2), and four racial–ethnic groups (Supplementary Table S2). These four ethnic groups presented different polymorphism patterns for these five genes. We checked the Hardy–Weinberg equilibrium (HWE) analysis of these five genes to assess the distribution equilibrium of the evolutionary mechanisms in population genetics [82], associated with factors such as population migration or stratification and disease association. *MTRR* A66G had significant (*p* < 0.05) HWE with disequilibrium for the Hispanic subgroup. We further checked the distribution of alleles for population-based allele frequencies across the ethnic groups to provide the reference distribution to our findings (Supplementary Table S2). To decrease the degrees of freedom and increase the power in the statistical testing, the total polymorphism score was recoded into two groups using the median split between <4 and ≥4. Increased polymorphism of the five genes combined was associated with a trend for increased risk of CRC (*p* < 0.1) (Table 2).

Table 3 presents the descriptive statistics of metabolites among four groups. Homocysteine and MMA levels were higher in the cancer group than the health controls (both *p* < 0.05), with homocysteine increasing incrementally along the disease groups. MMA levels were highest in the early cancer group than the two control groups (both post-hoc *p* < 0.05). Cystathionine (a converted metabolite from homocysteine through metabolism) was higher in two of the early-stage disease groups for both control and cancer groups (*p* < 0.05, for all groups and two post-hoc tests) with early cancer stage group presenting the highest value.

For various metabolites including methionine, methyl-folate, and betaine, there were downward trends for these metabolites along the disease groups from most healthy to most advanced disease groups. The difference on the metabolites among four racial groups are presented in Supplementary Table S3. Noteworthy significant findings included that Caucasian and Hispanic groups presented higher SAM/SAH ratio (a global indicator of methylation status) than the Asian and African; Betaine (helps body metabolize homocysteine) being highest in Asian and lowest in Hispanic groups; and B6 being highest in Caucasian than other three groups (all *p* < 0.05).

### 3.2. Most Influential Factors—The Ensemble Method

Supplementary Table S4 presents the most influential factors among three domains of genetic parameters (Supplementary Table S4a), metabolites (Supplementary Table S4b), top ranked demographic and lifestyle parameters (Supplementary Table S4c), using the bootstrap prediction modeling. The most crucial genetic predictor of cancer (Supplementary Table S4a) appeared to be the total polymorphism-mutations of all five genes. On the rank order of importance on the metabolites (Supplementary Table S4b), homocysteine and MMA ranked the highest. And, among the top demographic and lifestyle parameters, age ranked as the most significant parameter (Supplementary Table S4c). And, the most significant parameters for all three domains included homocysteine, age, total mutations of five genes, methyl-folate, MMA, and vegetable intake (Supplementary Table S4d).

### 3.3. Predictive Modeling for Healthy Eating—Generalized Regression Analysis

**Figure 1:**
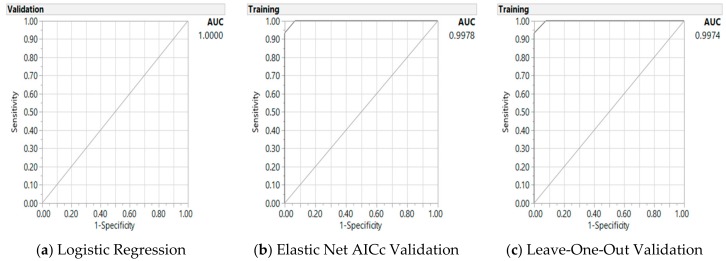
Receiver operating characteristic curve and area under the curve (AUC) for baseline logistic regression model (**a**) and generalized regression Elastic Net with Akaike’s information criterion with corrections (AICc) validation model (**b**) and leave-one-out validation model (**c**) on the predictors of colorectal cancer from gene-metabolite interaction, with one interaction term.

Using the most influential variables identified in earlier section, two GR models were developed using the Elastic Net GR models of AICc and LOO validation methods to predict the probability of cancer. In each case, the models were first compared to a LR model with validation for a baseline. The parameter estimates along with the associated *p*-values for the baseline LR results with validation are shown in the left panels of Table 4. The regularized parameters remaining in the GR elastic net AICc and LOO models are shown in the right panels of Table 4. The predictive performance for the GR Elastic Net models can be characterized by examining the misclassification rates, AICc, and AUC (Figure 1).

For the prediction of CRC with genes, metabolites, and demographic/lifestyle parameters, the most influential predictors included metabolites of homocysteine (7 µmol/L), methyl-folate (40 nmol/L); total gene mutations (≥4); age (51 years) and vegetable intake (2 cups) for demographic/diet parameters (Table 4); and interactions of gene mutations and MMA metabolite (400 nmol/L), all parameters except MMA being significant with the GR models (*p* < 0.0001 for AICc validation model, and *p* < 0.0001 for the interaction term and homocysteine and other parameter *p* < 0.05 for LOO cross validation model). MMA as an individual parameter must remain in the model because of its interaction with another parameter, total gene mutation. However, none of these parameters were significant with the LR model. Misclassification rates for these three methods were at 20% for LR, 3% for AICc and 4% for LOO GR models. AICc was 27 for LR and 26 for GR AICc model. And AUC was close to 100% for all three models (see Figure 1). Therefore, GR models outperformed LR model in the prediction of cancer status based on gene-metabolites interaction. 

**Figure 2:**
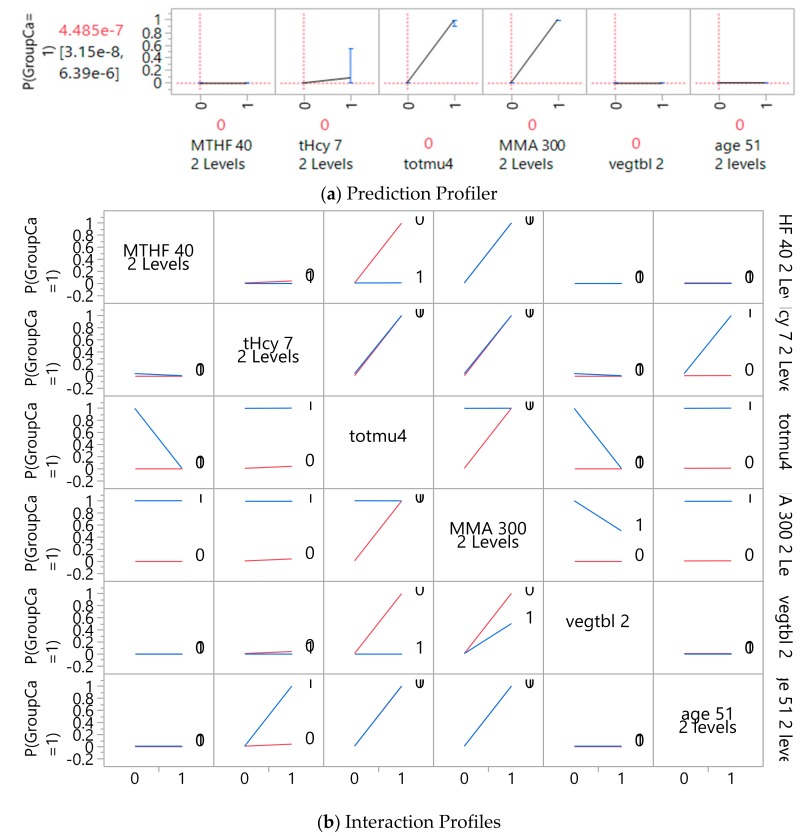
Prediction profiler (**a**) for significant predictors of colorectal cancer, and (**b**) interaction profiles of included parameters. Note. Non-parallel lines denote interactions between parameters in association with probability of cancer status (*p* (GroupCa = 1)), predictive parameters coded in 2 levels by median values; MTHF 40: Methyl folate level 40 nmol/L; tHCY 7: Total homocysteine 7 µmol/L; totmu4: total gene mutation score ≥4; MMA 300: Methylmalonic acid 300 nmol/L; vegtbl 2: Vegetable intake 2 cups.

The prediction profiler shown in Figure 2a and Supplementary Figure S1a, and the interaction profiler shown in Figure 2b and Supplementary Figure S1b, are illustrative of how to interpret the interaction results. To illustrate, in Figure 2, the excerpt of the interaction profiler depicts interactions between total gene mutations and MMA with apparent non-parallel lines in association with the prediction of cancer status (*p* (GroupCa = 1): Probability of predicting cancer status, 1 being yes). Visually, the more non-parallel the two levels, the more likely there is a significant interaction between the two parameters. For example, we see non-parallel lines for the total gene mutations with MMA, but also with MTHF and vegetable intake. In Supplementary Figure S1, the profilers and interaction profilers for the gene parameters are presented. No apparent interactions are present except for the total gene mutations with *DHFR* 19 bp deletion. However, further GR tested models did not present significant findings.

**Figure 3:**
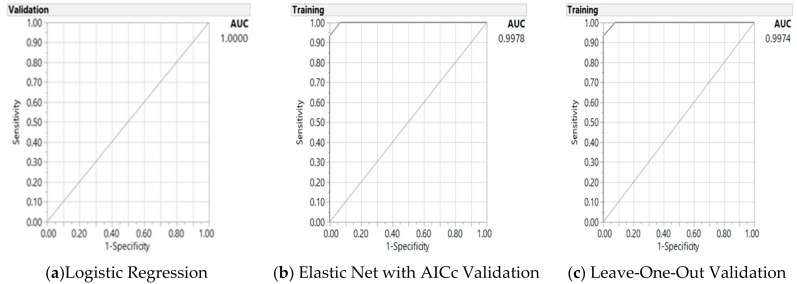
Receiver operating characteristic curve and AUC for baseline logistic regression model (**a**) and generalized regression Elastic Net AICc validation model (**b**) and leave-one-out validation model (**c**) on the predictors of colorectal cancer from gene-metabolite interactions, with two interaction terms.

As appeared in Figure 2, we further tested the interaction terms of MTHF and vegetable intake with total gene mutations in the GR models, however without significance. In a similar way to the previous model in Table 4, in the second model we added the interaction term of total gene mutation and MTHF (Table 5). This second model with one additional interaction term presented about the same level of statistical significance on the parameters’ estimates using the GR validation models, and similar misclassification rates and AUCs (Figure 3). This additional interaction term, however, was not significant and was left out of the model using the LOO validation method. In addition, the AICc were slightly larger (30 as compared to 26 for GR AICc and 27 for LR models) for less fit than the previous model in Table 4.

## 4. Discussion

We presented the genes and metabolites in the OCM pathway and their interactions on the prediction of CRC with dietary lifestyle factors by using various machine-learning based analytics to validate the findings across the methods. As a proof-of-concept study to examine genes and metabolites in the OCM pathway for cancer prevention, we used the ensemble method, as it is a well-known remedy for small-sample studies to validate the analyses by the random subsets of samples [68]. We further used GR method integrating significant parameters and bivariate interactions to maximize the model quality with the simplest optimal model. While previous studies have presented gene–environment interactions, associating genes in the OCM with folate deficiency [39,40,42] and CRC [39,42], new predictive modeling and validation analytics with interactions have become readily available for convenient use through SAS JMP programming (SAS Institute, Cary, NC, USA). Therefore, we included the genes and metabolites, to examine potential epigenetic mechanisms. Overall, the CRC group had higher homocysteine and MMA levels, lower methyl-folate, and increased combined gene polymorphisms for five genes in the OCM pathway than the control group. Additional modifiable factors included dietary intakes of vegetables for CRC risks. In a previous study [43], we presented the GR models for gene-environment interactions including these five genes interacting with environmental and lifestyle factors. It is noteworthy to point out that while we included only 30 cases in this study, the accuracy of prediction with the gene-metabolites are much better than the models with gene-environment factors as presented before [43] that included more cases, with lower misclassification rates (3–20% versus 28–34%), validated with lower AICc (26–30, the lower the better), and much higher AUC (99% versus 75–76%).

We presented the very first study cross-validating the effects of metabolites and genes along with healthy intakes of vegetables using both conventional LR inferential statistics and new methods including the ensemble method to handle multi-dimensional factors to predict the risk of CRC. While there are limitations to family-based, case-control designs because of genetic associations among the family members, we used the family-based analysis technique to explore and control for the family associations. Despite these limitations, methodological advantages for family-based studies by including family members can enforce the active participation of the family as an ecological unit, and more reliable reporting of lifestyle parameters [83,84], with a heightened awareness within the family unit to adopt healthier lifestyles. Thus, the rigor and reliability of the data are enhanced for sustainable interventions with lifestyle improvements. With a small sample size of 30 cases, the findings from this study need to be interpreted with caution. While we used both ensemble method and GR methods that are suitable for small sample sizes [67,68], further studies are needed to include larger samples to further validate these findings for various ethnic groups.

To add to the genetic factors, our results point to healthy dietary intakes as modifiable lifestyle factors [39,41,53] in relation to the gene–metabolite interactions for the prevention of CRC. The top modifiable factors included dietary intakes of vegetables, fruits, and grains, which are major food sources for healthy dietary fibers. The *MTHFR* gene is known to be associated with many chronic diseases, including CRC [12,13,14]. And, *MTHFR* and other genes in the OCM pathway play important roles in DNA methylation, a key mechanism in epigenetics, and more specifically nutrigenomics within the OCM pathway. Studies have emerged to document the effects of low folate levels and increased CRC risk [14,15,16]. The mechanism of low folate levels and CRC as well as a plethora of major cardiovascular and neurodevelopmental diseases have been associated with the toxic effect of hyperhomocysteinmia [12,14,15,16]. Supplementations of B9 and B12 nutrients along with the monitoring of these enzymes-metabolites including MMA and homocysteine levels were recommended for the cancer and aging populations when neurological pain/dysfunctions and functional deteriorations of multiple body systems occur [29,32]. While nutrient supplementation was questioned for potential harmful effects in molecularly heterogeneous CRC subtypes, postdiagnostic supplementation of methyl donor nutrients and alcohol did not affect the risk of death for nonmetastatic CRC in large epidemiologic studies [85]. In addition, previous studies presented the associations of increased homocysteine levels with microsatellite instability (MSI) in CRC case-only design (no control group) [86], and MSI with *MTHFR* 677 TT genotype [87]. Both *MTHFR* 677 TT genotype and increased homocysteine levels can lead to methyl donor deficiency that can increase MSI, particularly for aging populations [87]. Additionally, lower concentrations of nutrients related to the OCM pathway, such as folate and B vitamins (B6, B12, B2), led to elevated homocysteine levels, which decreased OCM pathway activities for epigenetic mechanisms. Hence, insufficient methyl groups in the diet and blood levels compromised DNA methylation, synthesis or repair, thus potentially promoted carcinogenesis [15,88], concluded by the meta-analyses for CRC [15,89].

Recent studies including meta-prediction studies that examined gene–environment interactions consistently presented that environmental factors such as air pollution being associated with increased gene polymorphism and trends to increased disease risks across various disease conditions, especially for *MTHFR* C677T polymorphisms and genes in the methylation pathways [11,90,91,92,93,94,95]. Environmental toxicants such as air pollution and smoking can induce oxidative stress and dis-regulate reactive oxygen species that causes damage to cellular DNA that leads to mutations, genomic instability, and ultimately malignancy [90,91,92]. To mitigate these effects, we demonstrated in our study that healthy intake on vegetables and grains, working in synergy with enzyme metabolites in the OCM pathway, are helpful to detox by reducing homocysteine toxicity, to prevent CRC. From these understandings, future studies may focus on the epigenetics of methyl-donors and fibers to detox the hazards from inflammatory processes, with healthy lifestyles to prevent CRC. Additionally, future research can be designed to continue with the examination of healthy lifestyles with gene–environment interactions to prevent cancer.

## Figures and Tables

**Table 1:** Comparison on demographic factors between control and cancer groups.

Factors	Control (Groups 1, 2)		Cancer (Groups 3, 4)		
*n* (%) or M ± SD (Ranges)	1-Healthy	2-Chronic Diseases	3-Cancer	4-Advanced	*p*
	(*n* = 4)	(*n* = 11)	(*n* = 5)	(*n* = 10)	
Gender					
Male	0 (0%)	4 (36.4%)	5 (100%)	2 (20%)	0.008
Female	4 (100%)	11 (63.6%)	0 (0%)	8 (80%)	
Age (Years)	34 ± 14	43 ± 12	50 ± 11	60 ± 9	0.006
	(19–51)	(21–58)	(38–62)	(44–72)	
Posthoc	<4 (*p* = 0.013)	<4 (*p* = 0.048)			
BMI	24 ± 3.2	28 ± 8.5	24 ± 2.2	31 + 8.6	0.24
	(17–28)	(21–49)	(19–29)	(19–51)	
Weight (Kg)	63 ± 6.8	77 ± 26	72 ± 11	79 + 26	0.59
	(57–71)	(52–141)	(59–88)	(45–138)	
Vegetable intake	2.3 ± 0.0	2 ± 0.8	2.6 ± 0.6	1.6 ± 0.7	0.087
Cup Servings	(2–3)	(1–3)	(2–3)	(1–3)	
Posthoc				<3 (*p* = 0.027)	
Fruit	1.3 ± 1.0	1.5 ± 0.7	1.8 ± 0.5	0.9 ± 0.7	0.073
Cup Servings	(0–2)	(0–2)	(1–2)	(0–2)	
Posthoc				<3 (*p* = 0.015)	
Whole grain cups	1.5 ± 0.6	1.7 ± 0.7	1.8 ± 0.8	1.8 ± 0.8	0.92
	(1–2)	(1–3)	(1–2)	(0–2)	
Liquid cups	5.8 ± 1.5	5.5 ± 1.6	6.2 ± 1.6	5.3 ± 1.5	0.56
	(5–8)	(4–8)	(5–8)	(4–8)	
Race					
White (10)	1 (25%)	3 (27.3%)	2 (20%)	4 (40%)	0.68
Asian (9)	2 (50%)	3 (27.3%)	3 (30%)	1 (10%)	
Hispanic (9)	1 (25%)	4 (36.4%)	0 (0%)	4 (40%)	
African (2)	0 (0%)	1 (9.1%)	0 (0%)	1 (10%)	

Nonparametric test, Posthoc by Wilcoxon test. 4 groups: Inflammation status indicated by chronic health diseases (Group 2) or advanced cancer stage (Group 4); M: median; SD: standard deviation; BMI: body mass index.

**Table 2:** Comparisons on gene polymorphisms between control and cancer groups.

Genotype	Control (Groups 1, 2)		Cancer (Groups 3, 4)		*p*
Enzyme Deficiency	1-Healthy	2-Chronic Disease	3-Cancer	4-Advanced	
	(*n* = 4)	(*n* = 11)	(*n* = 5)	(*n* = 10)	
*MTHFR* 677					
0 (CC)	2 (50%)	5 (45.4%)	2 (40%)	2 (20%)	0.70
1 (CT)	1 (25%)	5 (45.4%)	2 (40%)	7 (70%)	
2 (TT)	1 (25%)	1 (9.1%)	1 (20%)	1 (10%)	
*MTHFR* 1298					
0 (AA)	2 (50%)	7 (63.6%)	4 (80%)	7 (70%)	0.82
1 (AC)	2 (50%)	4 (36.4%)	1 (20%)	2 (20%)	
2 (CC)	0 (0%)	0 (0%)	0 (0%)	1 (10%)	
*MTR* 2756					
0 (AA)	2 (50%)	7 (63.6%)	4 (80%)	3 (30%)	0.40
1 (AG)	2 (50%)	2 (18.2%)	1 (20%)	6 (60%)	
2 (GG)	0 (0%)	2 (18.2%)	0 (0%)	1 (10%)	
*MTRR* 66					
0 (AA)	2 (66.7%)	6 (54.5%)	4 (40%)	0.93
1 (AG)	0 (0%)	3 (27.3%)	1 (20%)		4 (40%)
2 (GG)	1 (33.3%)	2 (18.2%)	1 (20%)		2 (20%)
*DHFR* 19					
00 (++)	1 (25%)	5 (45.4%)	0 (0%)	3 (30%)	0.69
01 (+−)	2 (50%)	4 (36.4%)	2 (40%)	4 (40%)	
11 (−−)	1 (25%)	2 (18.2%)	3 (60%)	3 (30%)	
Total Mutation					
≥4	1 (25%)	4 (36.4%)	1 (20%)	8 (80%)	0.077
	3.25 ± 0.50	3.36 ± 1.57	2.20 ± 1.30	3.90 ± 1.45	0.16
	(3–4)	(1–6)	(1–4)	(1–6)	
Posthoc			<4 (*p* = 0.049)		

Nonparametric test, Posthoc by Wilcoxon test. 4 groups: Inflammation status indicated by chronic health diseases (Group 2) or advanced cancer stage (Group 4). *MTHFR: methylenetetrahydrofolate; MTR: methionine synthase; MTRR: methionine synthase reductase; DHFR*: *dihydrofolate reductase*.

**Table 3:** Comparisons on metabolites in the blood plasma among control and cancer groups.

Metabolites	Control (Groups 1, 2)		Cancer (Groups 3, 4)		*p*
M + SD (ranges)	1-Healthy *n* = 4	2-Chronic Disease *n* = 11	3-Cancer *n* = 5	4-Advanced *n* = 10	
Homocysteine (µmol/L)	4.5 ± 1.8 (3.1–7)	5.1 ± 1.0 (4.2–7.2)	8.6 ± 3.8 (5.8–14)	9.1 ± 4.2 (4–17)	0.014
Posthoc	<4 (*p* = 0.023)	<3 (*p* = 0.028)<4 (*p* = 0.019)			
SAM (nmol/L)	85 ± 24 (70–122)	89 ± 17 (63–120)	129 ± 61 (77–233)	102± 21 (67–134)	0.12
SAH (nmol/L)	25 ± 14 (11–43)	23 ± 7.2 (12–38)	52 ± 51 (23–142)	29 + 13 (16–56)	0.25
Posthoc		<3 (*p* = 0.041)			
SAM/SAH Ratio	4.1 ± 1.9 (1.7–6.3)	4.2 ± 1.1 (2.8–6.3)	3.2 ± 1.1 (1.6–4.6)	3.9 ± 1.1 (0–5.2)	0.56
ADMA (nmol/L)	573 ± 198 (393–849)	519 ± 110 (278–720)	666 ± 223 (472–917)	557 ± 110 (406–754)	0.77
SDMA (nmol/L)	488 ± 130 (324–642)	466 ± 78 (340–589)	885 ± 671 (401–2050)	516 ± 109 (425–778)	0.44
Methionine (nmol/L)	37 ± 10 (27–51)	30 ± 7.3 (20–46)	32 ± 4.8 (26–39)	26 ± 6.2( 18–38)	0.14
Posthoc		<3 (*p* = 0.041)			
MMA (nmol/L)	249 ± 48 (185–301)	285 ± 229 (178–972)	359 ± 72 (304–480)	274 ± 97 (186–521)	0.025
Posthoc	<3 (*p* = 0.02)	<3 (*p* = 0.013)			
Cystathionine (nmol/L)	423 ± 267 (227–796)	243 ± 147 (107–600)	470 ± 221 (193–692)	244 ± 102 (149–502)	0.043
Posthoc		<3 (*p* = 0.041)		<3 (*p* = 0.043)	
Betaine (nmol/L)	71 ± 18 (48–89)	63 ± 20 (38–111)	61 ± 24 (37–96)	53 ± 11 (36–67)	0.45
Vitamin B-6 (nmol/L)	50 ± 16 (29–67)	60 ± 42 (14–155)	64 ± 52 (5.3–128)	46 ± 24 (20–88)	0.95
5-MTHF (nmol/L)	30 ± 10 (18–43)	48 ± 19 (30–97)	36 ± 5.3 (32–45)	36 ± 16 (18–78)	0.063
Posthoc				<2 (*p* = 0.045)	
Choline (nmol/L)	12 ± 5.7 (7.9–21)	9.7 ± 2.8 (5.7–16)	14 ± 7.5 (8–27)	10 ± 3.1 (6.9–18)	0.50

Nonparametric test, Posthoc by Wilcoxon test; 4 groups: inflammation status indicated by chronic health diseases (Group 2) or advanced cancer stage (Group 4); SAM: *S*-adenosylmethionine; SAH: *S*-adenosylhomocysteine; ADMA: Asymmetric dimethylarginine; SDMA: Symmetric dimethylarginine; MMA: Methylmalonic acid; 5-MTFH: 5-methyltetrahydrofolate or methyl-folate.

**Table 4:** Baseline logistic regression model and generalized regression elastic net models on the prediction of colorectal cancer from gene-metabolite interaction, with one interaction term.

	Logistic Regression Original Model		Generalized Regression Elastic Net Model			
			AICc Validation		Leave-One-Out Validation	
Parameters	Estimate	*p* (*X*^2^)	Estimate	*p* (*X*^2^)	Estimate	*p* (*X*^2^)
(Intercept)	−5.6	0.93	0.4	0.78	1.1	0.45
MMA * Gene mutations	−42	0.68	−30	<0.0001	−11	<0.0001
Homocysteine	−15	0.77	−12	<0.0001	−5.7	<0.0001
Methyl-folate	14	0.69	9.1	<0.0001	3.4	0.0019
Gene mutations	14	0.86	11	<0.0001	4.0	0.0188
Vegetable intake	28	0.62	17	<0.0001	5.6	0.0005
Age	−14	0.63	−8.7	<0.0001	−2.9	0.0024
MMA	−0.4	0.996	−1.7	0.28	0	1.0
Misclassification Rate	0.2	–	0.03	–	0.04	–
AICc	27	–	26	–	–	–
Area under the curve	1.0	–	0.998	–	0.997	–

MMA: Methylmalonic acid; *: Interaction; –: Not available; AICc: Akaike’s information criterion with corrections: AUC: Area under the curve.

**Table 5:** Baseline logistic regression model and generalized regression Elastic Net models on the prediction of colorectal cancer from gene-metabolite interactions, with two interaction terms.

	Logistic Regression Original Model		Generalized Regression Elastic Net Model			
			AICc Validation		Leave-One-Out Validation	
Parameters	Estimate	*p* (*X*^2^)	Estimate	*p* (*X*^2^)	Estimate	*p* (*X*^2^)
(Intercept)	−0.4	0.997	−0.36	0.79	1.2	0.38
MMA * Gene mutations	−35	0.77	−29	<0.0001	−9.2	<0.0001
Homocysteine	−13	0.63	−12	<0.0001	−4.9	<0.0001
Methyl-folate (MTHF)	10	0.48	8.7	<0.0001	2.8	0.0093
Gene mutations ≥4	17	0.92	12	0.0007	3.2	0.0496
Vegetable intake	20	0.35	16	<0.0001	4.4	0.0033
Age	−10	0.45	−8.1	<0.0001	−2.5	0.0096
MMA	−1.9	0.99	−0.7	0.64	0	1.0
MTHF * Gene mutations	−4.0	0.98	−0.2	0.92	0	1.0
Misclassification Rate	0.03	–	0.03	–	0.04	–
AICc	30	–	30	–	–	–
Area under the curve	0.998	–	0.998	–	0.997	–

MMA: Methylmalonic acid; *: Interaction; –: Not available; AICc: Akaike’s information criterion with corrections: AUC: Area under the curve.

## Supplementary Materials

The following are available online at http://www.mdpi.com/2075-4426/8/3/26/s1, Table S1: Comparisons on demographic factors across racial groups; Table S2: Distribution of gene polymorphisms per control and cancer groups across racial groups; Table S3: Comparisons on plasma metabolites among racial groups. Supplementary Table S4. Bootstrap forest analysis of three domains and significant parameters included in the prediction model: (a) gene parameters; (b) metabolites; (c) top demographic and lifestyle parameters; (d) most significant parameters of three domains. Figure S1: Gene parameters: total gene mutation >4 (totmu4), MTHFR C677T, MTHFR A1298C, *MTR* A2756G, *MTRR* A66G, *DHFR* 19bp deletion, and MTHFR deficiency >50% calculated from *MTHFR* 677 T and 1298 C alleles: (a) prediction profiler; (b) examples of interaction profiles on DHFR 19 bp deletion interacting with total gene mutation >4 in association with probability of predicting cancer (*p* (GroupCa = 1)).
