# Supplementary material for: Gene-Metabolite Interaction in the One Carbon Metabolism Pathway: Predictors of Colorectal Cancer in Multi-Ethnic Families
Source: J Pers Med. 2018 Aug 6;8(3):26. doi: 10.3390/jpm8030026 (PMC6164460; doi:10.3390/jpm8030026)
Supplement: Supplementary file 1 [file jpm-08-00026-s001.pdf]

Supplementary File:

Gene-Metabolites Interaction in the One Carbon Metabolism Pathway: Predictors of Colorectal Cancer in Multi-Ethnic Families

S. Pamela K. Shiao*, James Grayson, Chong Ho Yu
*Corresponding: pshiao@msn.com

Supplementary Table S1. Comparisons on demographic factors across racial groups. Nonparametric test, Posthoc by Wilcoxon test.

		**White** (n = 10)	**Hispanic** (n = 9)	**Asian** (n = 9)	**African** (n = 2)	p
Gender	male	4 (40%)	2 (22%)	4 (44%)	1 (50%)	0.77
	female	6 (60%)	7 (78%)	5 (56%)	1 (50%)	
Age	years	48 ± 15	50 ± 14	47 ± 13	52 ± 25	0.98
		(21 – 70)	(29 – 72)	(19 – 62)	(34 – 69)	
BMI		30 ± 8.7	28 ± 6.5	23 ± 2.1	39 ± 11	0.036
		(21 – 51)	(21 – 43)	(21 – 27)	(31 – 47)	
	posthoc			<White p = 0.03		
				<African p = 0.045		
Weight	Kg	80 ± 26	73 ± 15	63 ± 6.3	113 ± 38	0.035
		(45 – 138)	(54 – 109)	(52 – 71)	(86 – 141)	
	posthoc			<African p = 0.045		
Vegetable	cups	2.1 ± 0.2	1.8 ± 0.2	2.3 ± 0.2	1.0 ± 0.5	0.093
		(1 – 3)	(1 – 1)	(1 – 3)	(1 – 1)	
Fruit	cups	1.2 ± 0.8	1.1 ± 0.8	1.6 ± 0.5	1.5 ± 0.7	0.59
		(0 – 2)	(0 – 2)	(1 – 2)	(1 – 2)	
Whole grain	oz	2.2 ± 0.6	1.6 ± 0.5	1.4 ± 0.7	1.5 ± 0.7	0.071
		(1 – 3)	(1 – 2)	(1 – 3)	(1 – 2)	
	posthoc		<White p = 0.029	<White p = 0.038		
Liquid	cups	5.5 ± 1.4	6.1 ± 1.8	5.4 ± 1.5	4.5 ± 0.7	0.59
		(4 – 8)	(4 – 8)	(4 – 8)	(4 – 5)	

BMI: body mass index.

Supplementary Table S2. Distribution of gene polymorphisms per control and cancer groups across racial groups.

n (%)	Control Group					Cancer Group		
**Genotypes**	**0**	**1**	**2**	***p* (HWE)**	**Allele Frequency#**	**0**	**1**	**2**
MTHFR 677	CC	CT	TT		% C/T	CC	CT	TT
Total	7 (47)	6 (40)	2 (13)	NS	75/25	4 (27)	9 (60)	2 (13)
White	0 (0)	5 (83)	1 (17)	NS	53/47	1 (25)	2 (50)	1 (25)
Asian	2 (50)	1 (25)	1 (25)	NS	70/30	3 (60)	1 (20)	1 (20)
Hispanic	1 (25)	3 (75)	0 (0)	NS	55/45	2 (40)	3 (60)	0 (0)
Black	1 (100)	0 (0)	0 (0)	–	91/9	1 (100)	0 (0)	0 (0)
MTHFR 1298	AA	AC	CC		% A/C	AA	AC	CC
Total	9 (60)	6 (40)	0 (0)	NS	75/25	11 (73)	3 (20)	1 (7)
White	5 (83)	1 (17)	0 (0)	NS	85/15	3 (75)	1 (25)	0 (0)
Asian	3 (75)	1 (25)	1 (25)	NS	78/22	3 (60)	2 (40)	0 (0)
Hispanic	2 (50)	1 (25)	1 (25)	NS	84/16	2 (40)	3 (60)	0 (0)
Black	1 (100)	0 (0)	0 (0)	–	85/15	1 (100)	0 (0)	0 (0)
MTR 2756	AA	AG	GG		% A/G	AA	AG	GG
Total	9 (60)	4 (27)	2 (13)	NS		7 (46.7)	7 (46.7)	1 (6.7)
White	2 (33)	3 (50)	1 (17)	NS	84/16	2 (50)	1 (25)	1 (25)
Asian	2 (50)	2 (50)	0 (0)	NS	65-91/9-35	2 (40)	2 (40)	1 (20)
Hispanic	3 (75)	1 (25)	0 (0)	NS	19/81	5 (100)	0 (0)	0 (0)
Black	0 (0)	1 (100)	0 (0)	NS	30-37/63-70	0 (0)	1 (100)	0 (0)
MTRR 66	AA	AG	GG		% A/G	AA	AG	GG
Total	8 (57)	3 (21)	3 (21)	NS	64/36	7 (47)	5 (33)	3 (20)
White	2 (33)	3 (50)	1 (17)	NS	45/55	0 (0)	1 (25)	3 (75)
Asian	2 (50)	1 (25)	1 (25)	NS	74/26	2 (50)	2 (50)	0 (0)
Hispanic	3 (75)	0 (0)	1 (25)	0.0455	72/28	5 (100)	0 (0)	0 (0)
Black	0 (0)	1 (100)	0 (0)	NS	73/27	1 (0)	0 (0)	0 (0)
DHFR 19 bp	II	ID	DD		% I/D	II	ID	DD
Total	6 (40)	6 (40)	3 (20)	NS	50/50	3 (20)	6 (40)	6 (40)
White	1 (17)	3 (50)	2 (33)	NS	45-47/53-55	2 (50)	0 (0)	2 (50)
Asian	0 (0)	2 (50)	2 (50)	NS	63/37	2 (40)	3 (60)	0 (0)
Hispanic	1 (25)	1 (25)	2 (50)	NS	58/42	1 (20)	3 (60)	1 (20)
Black	1 (100)	0 (0)	0 (0)	–	55/45	1 (100)	0 (0)	0 (0)

HWE: Hardy-Weinberg Equilibrium; *MTHFR: methyelene tetrahydrofolate reductase; MTR: methionine synthase; MTRR: methionine synthase reductase; DHFR: dihydrofolate reductase;* NS: Not significant; --: cannot be calculated; HWE Calculator: http://www.bioinformatics.org/forums/forum.php?forum_id=3196; #Population Allele Frequencies from: http://useast.ensembl.org/index.html; https://www.cdc.gov/genomics/population/genvar/frequencies/mthfr.htm.

Supplementary Table S3. Comparisons on metabolites in blood plasma among racial groups.

	White (n = 10)	Hispanic (n = 9)	Asian (n = 9)	African (n = 2)	*p*
Homocysteine	8.3 ± 4.5	6.6 ± 3	6.2 ± 3.3	5.6 ± 0.4	0.72
µmol/L	(4.2 – 17)	(3.2 – 12)	(3.1 – 14)	(5.3 – 5.9)	
SAM	99 ± 20	94 ± 16	108 ± 53	93 ± 1.2	0.96
nmol/L	(74 – 134)	(70 – 125)	(63 – 233)	(92 – 94)	
SAH	27 ± 12	21 ± 5.6	41 ± 39	38 + 14	0.17
nmol/L	(11 – 56)	(12 – 29)	(16 – 142)	(27 – 47)	
SAM/SAH Ratio	4.1 ± 1.1	4.7 ± 1.0	3.2 ± 1.0	2.7 ± 1.0	0.031
	(2.4 – 6.3)	(3.4 – 6.3)	(1.6 – 4.4)	(2.0 – 3.4)	
Posthoc			< Hispanic $p = 0.012$		
ADMA	531 ± 89	626 ± 232	534 ± 92	574 ± 9.2	0.57
nmol/L	(435 – 754)	(278 – 917)	(393 – 697)	(567 – 580)	
SDMA	503 ± 96	700 ± 523	479 ± 133	511 ± 21	0.58
nmol/L, -324	(401 – 686)	(349 – 2050)	(324 – 778)	(496 – 526)	
Methionine	28 ± 5.3	29 ± 6.6	34 ± 10	28 ± 5.1	0.49
nmol/L	(18 – 35)	(18 – 38)	(20 – 51)	(24 – 31)	
MMA	288 ± 98	276 ± 96	322 ± 246	199 ± 18	0.31
nmol/L	(178 – 521)	(185 – 480)	(205 – 972)	(186 – 211)	
Betaine	56 ± 10	49 ± 12	78 ± 20	60 ± 1.4	0.0098
nmol/L	(37 – 68)	(36 – 75)	(47 – 111)	(59 – 61)	
Posthoc		<White $p = 0.016$			
		< Asian $p = .0047$			
Vitamin B-6	82 ± 40	46 ± 30	41 ± 20	22 ± 2.9	0.028
nmol/L	(29 – 155)	(14 – 109)	(5.3 – 67)	(20 – 24)	
Posthoc		<White, $p = .025$	<White, $p = .0455$	<White, $p = .041$	
5-MTHF nmol/L	45 ± 15	40 ± 24	34 ± 7.4	41 ± 1.6	0.24
	(31 – 78)	(18 – 97)	(25 – 48)	(40 – 43)	
Choline	9.7 ± 3.2	9.7 ± 1.7	13 ± 6.6	9.9 ± 0.8	0.59
nmol/L	(5.7 – 18)	(7.4 – 12)	(6.9 – 27)	(9.3 – 10)	

Nonparametric test, Posthoc by Wilcoxon test. SAM: *S*-adenosylmethionine; SAH: *S*-adenosylhomocysteine; ADMA: Asymmetric dimethylarginine; SDMA: symmetric dimethylarginine; MMA: Methylmalonic acid; 5-MTFH: 5-methyltetrahydrofolate or methylfolate.

Supplementary Table S4. Bootstrap forest analysis of three domains and significant parameters included in the prediction model: (a) gene parameters, (b) metabolites, (c) top demographic and lifestyle parameters, (d) most significant parameters of three domains.

(a) Gene parameters

Term	Number of Splits	G^2	Portion
Total mutation > 4	16	0.34	0.22
DHFR 19bp del	23	0.33	0.21
MTRR A66G	27	0.25	0.16
MTR A2756G	21	0.19	0.12
MTHFRd50	14	0.16	0.11
MTHFR C677T	12	0.16	0.10
MTHFR A1298C	16	0.13	0.08

(b) Metabolties

Term	Number of Splits	G^2	Column Contribution	Portion
Homocysteine 7	25	1.59		0.24
MMA 300	30	1.35		0.20
SAM 90	27	0.75		0.11
Betaineb 60	22	0.72		0.11
MTHF 40	19	0.48		0.07
Methionine 30	19	0.44		0.03
SAH 27	19	0.38		0.06
SDMA 400	9	0.29		0.04
Ratio SAM/SAH 4	15	0.23		0.03
Vitamin B6 55	14	0.15		0.02
Cystathionine 290	12	0.14		0.02
Choline 10	14	0.13		0.02
ADMA 400	1	0.03		0.004

(c) Top demographic and lifestyle parameters

Term	Number of Splits	G^2	Portion
age 51	25	1.53	0.52
fruit intake	30	0.40	0.13
Sex	28	0.39	0.13
BMI overweight	17	0.32	0.11
vegetable intake	16	0.14	0.05
Whole grain intake	16	0.12	0.04
liquid intake	18	0.06	0.02

(d) Most significant parameters of three domains

Term	Number of Splits	G^2	Portion
Homocysteine 7	28	1.85	0.35
age 51	26	1.23	0.23
Total mutation > 4	33	0.83	0.16
MTHF 40	32	0.71	0.13
MMA 300	14	0.57	0.11
Vegetable intake	24	0.09	0.02

Prediction Profiler

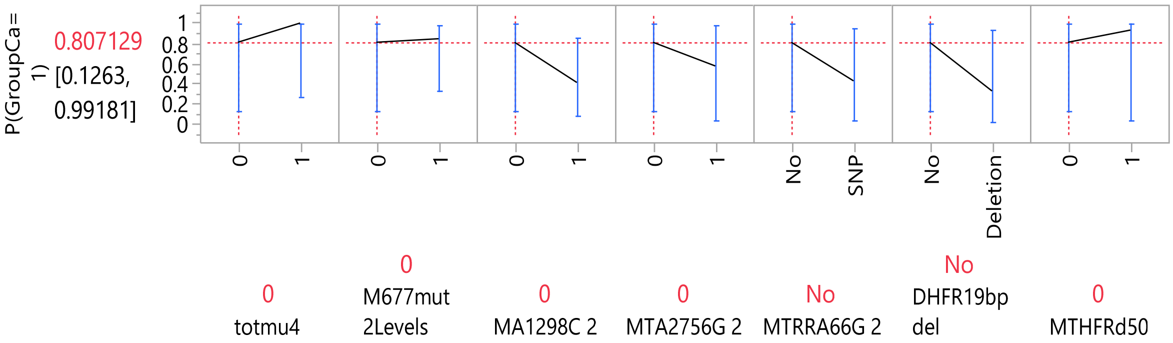

Interaction Profiles

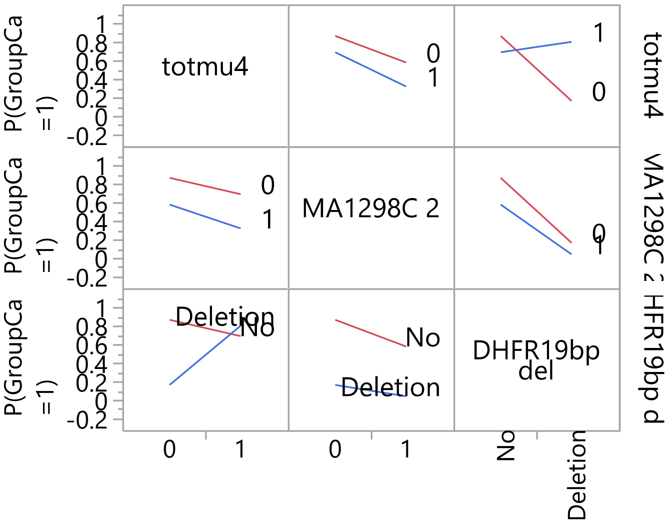

Supplementary Figure S1. Gene parameters: total gene mutation >4 (totmu4), *MTHFR* (*methyelene tetrahydrofolate reductase*) C677T, *MTHFR* A1298C, *MTR (methionine synthase)* A2756G, *MTRR (methionine synthase reductase)* A66G, *DHFR (dihydrofolate reductase)* 19 bp deletion, and MTHFR deficiency >50% calculated from *MTHFR* 677 T and 1298 C alleles: (a). prediction profiler, (b) examples of interaction profiles on *DHFR* 19 bp deletion interacting with total gene mutation > 4 in association with probability of cancer status [*p*(GroupCa=1)].
